# Infection prevention and control in the medical imaging environment: a scoping review

**DOI:** 10.1186/s13244-023-01470-1

**Published:** 2023-07-14

**Authors:** Yobelli A. Jimenez, Sarah J. Lewis

**Affiliations:** University of Sydney, Susan Wakil Health Building (D18), Western Avenue, Camperdown, NSW 2006 Australia

**Keywords:** Infection prevention and control, Medical imaging, SEIPS framework, Radiology, Computed tomography

## Abstract

**Abstract:**

Infection prevention and control (IPC) practices are key to preventing and controlling the spread of pathogens in medical imaging departments (MIDs). The objective of this scoping review was to synthesise information about current research in MID regarding IPC and to use the Systems Engineering Initiative for Patient Safety (SEIPS) model to identify the work system factors (‘persons’, ‘organisation’, ‘tools and technology’, ‘tasks’ and ‘environment’) influencing the practice of IPC, in order to better understand challenges and facilitators that affect IPC in MID. Predefined search terms and medical subject headings relating to IPC in the medical imaging setting were used to search 3 databases. A total of 46 publications met the inclusion criteria, which combined, encompassed all five SEIPS domains influencing IPC. The literature supports the interrelated nature of the five SEIPS domains, and influence to one another. Hand hygiene was a major focus of publications. Mechanisms of infection in contrast-enhanced computed tomography were most reported, with human error, lack of education, and issues associated with devices and processes mechanisms found to influence IPC breaches. A systems approach, such as the SEIPS model, is useful for understanding barriers and hence opportunities for improvement of IPC in the medical imaging setting. Future studies should address individuals’ decision-making processes in the medical imaging setting, and a greater focus should be placed into the procedural steps, education and tools used for contrast media administration.

**Critical relevance statement:**

A systems approach, such as the Systems Engineering Initiative for Patient Safety model, is useful for understanding barriers and hence opportunities for improvement of IPC in the medical imaging setting.

**Graphical Abstract:**

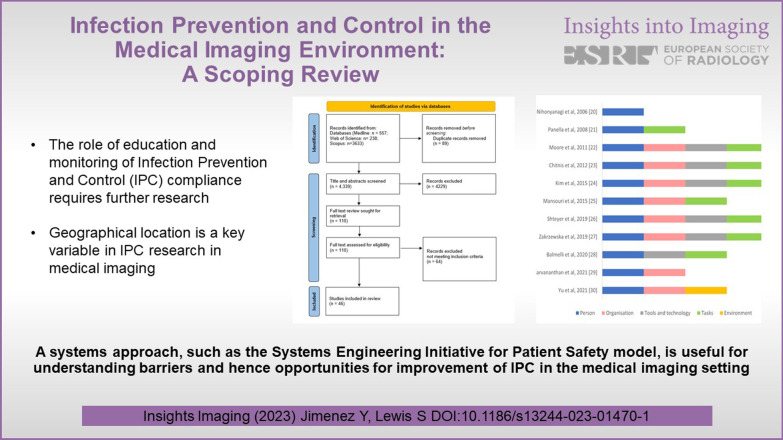

**Key points:**

1. IPC in the medical imaging setting would benefit from a systems approach.
2. The role of education and monitoring of IPC compliance requires further research.
3. Geographical location is a key variable in IPC research in medical imaging.

## Background

Medical imaging services are an integral component of the healthcare system [1]. In Australia and worldwide, medical imaging services are provided for diagnostic or treatment purposes in a range of settings, including public hospitals, private hospitals, and private practices, and are offered as out-patient and in-patient services. Risks of healthcare-associated infections in the radiology department were recently described by Ilyas et al., where contamination areas were identified in radiology equipment, medical devices and general usage areas [2]. This is supported by a recent systematic review by Picton-Barnes et al., which identified twelve studies describing infectious organisms present in diagnostic radiography departments, suggesting the need for improved infection control methods and/or compliance training to minimise infection risk [3]. Recommendations in opinion and commentary articles in medical imaging were frequently published after 2019, focusing on the COVID-19 pandemic [4–8]. The main topics in these publications were operational protocols used by radiology departments to safely image patients with suspected or confirmed COVID-19 diagnosis, and importance of hand hygiene.

Infection prevention and control (IPC) measures aim to prevent and control the spread of pathogens between people in healthcare settings. Whilst the importance of healthcare professionals adhering to IPC guidelines is well recognised, multiple studies report that healthcare professionals often fail to comply with standard precautions [9–11]. In the complex healthcare environment, it is expected that multiple factors impact healthcare professionals’ IPC practices. Factors may include an individual’s knowledge and behaviour, workplace culture and training, as well as the macro-work system in which an individual works. As such, the Systems Engineering Initiative for Patient Safety (SEIPS) model [12] provides a suitable framework for IPC practice descriptions in medical imaging. The SEIPS model has been used in health settings to identify deficiencies that can impair the delivery of high-quality care to patients, and focuses on healthcare structures, relationships, and processes for delivering patient-centred care [13, 14]. Specifically, the SEIPS model aims to describe the effects of a work system and process on health outcomes within five interrelated components: “persons”, “organisation”, “technologies and tools”, “tasks” and “environment” Sections [15]. In this model, an individual is the centre of the work system, the organisation consists of structures external to a person within which work is performed, tools and technologies are devices that are used to conduct tasks, tasks are specific actions within the larger work process, and the environment includes physical and safety environment factors [12, 16]. Table 1 describes how the SEIPS framework can be applied to medical imaging.

**Table 1:** Systems engineering initiative for patient safety (SEIPS) framework applied to medical imaging

SEIPS domain	Application to medical imaging
Person	• Radiographer, radiologist, radiology nurse, hospital staff, student practitioner • An individual’s education, skills and motivation
Organisation	• Structures external to an individual: MID regulations, policies, work schedules, hierarchy of supervision, and teamwork • The safety culture of the hospital or MID, where safety culture can be defined as the perceived priority that safety holds in the face of other competing demands, such as cost containment, efficiency, and reducing length of procedures
Tools and technology	• The objects that individuals use: medical imaging equipment, beds, PPE • An object’s usability, integration capabilities and maintenance requirements
Tasks	• Individual actions within the processes, such as performing medical imaging procedures, contrast administration, positioning and lifting a patient • Difficulty of tasks and time pressures whilst undertaking tasks
Environment	• The physical environment: physical layout of the medical imaging rooms, available space, air quality, lighting, noise, temperature • Work distractions and interruptions in MID

MID: Medical Imaging Departments; PPE: personal protective equipment

To our knowledge, the SEIPS model has not been used in the medical imaging field to identify work system factors influencing IPC. The objective of this scoping review was to synthesise information about current research methods used in medical imaging departments (MID) regarding IPC and to use the SEIPS model to identify the previously described work system factors influencing the practice of IPC. For this scoping review, MID encompass medical imaging clinical settings in public hospitals, private practice, as well as specific areas, such as general X-ray, computed tomography (CT) and magnetic resonance imaging (MRI). The review does not cover medical imaging that is conducted remotely to the core practice of imaging, such as where the responsibility for infection control within the “organisation” Section is not managed by a radiology department (e.g. day surgeries, operating theatres, cardiology units or sterile units).

## Methods

The scoping review was conducted in accordance with the framework for scoping reviews based on Joanna Briggs Institute methodology for conducting scoping reviews [17]. The Preferred Reporting Items for Systematic Reviews and Meta-analysis (PRISMA) reporting guideline extension for scoping reviews was followed [18].

### Inclusion and exclusion criteria

Studies that addressed staff working in the medical imaging setting, including radiographers, nurses, and radiologists, were included. Sonographers and staff working exclusively in sonography settings/practices were excluded. Studies that explored IPC were considered when they applied to individuals (patients and staff), team setting or organisational approaches. The concept of IPC aimed to encompass all measures that aim to prevent and control the spread of pathogens between people in the medical imaging setting, excluding sonography. This was primarily due to physical and practical differences between equipment used in sonography to those in other medical imaging settings. Other locations, where infection control was not managed by a radiology department, for example portable radiography in intensive care units (ICUs), were also excluded. The rationale for this is that these dedicated wards, such as ICU, are usually managed by a Nursing Unit Manager and may have specific requirements for IPC such as isolation rooms and reverse barrier care. In publications where interventions to improve IPC were described, they were only included when they focused on outcomes relating to knowledge, attitude and/or practice of IPC, and publications that focused solely on a description of the intervention itself were excluded. The scoping review considered all peer-reviewed publications that explored individuals’ attitudes, behaviour or practice relating to IPC in the medical imaging setting. Recommendations, editorial, opinions or commentary articles were excluded. Including all other study types, allowed an exploration of the scope of available literature, and to better understand how researchers approach investigation of IPC in the medical imaging context. Publications between 1992 and 2022 were included. Exclusion criteria applied to studies published in languages other than English, unless the abstract was available in English, which was noted as data collected from an abstract only. This was due to language limitations by authors and uncertainty of translation by automated methods.

### Search strategy

Searches were conducted on Medline, Web of Science, and Scopus for publications between 1992 and 2022 using the following search terms: attitude*, practice, knowledge, radiograph*, radiolog*, X-ray, computed tomography, CT, magnetic resonance imaging, MRI, infection control, infection prevention, infection prevention and control, contaminat*, combined with Boolean operators “AND” and “OR”. The final search was conducted in October, 2022.

### Screening and data charting process

**Fig. 1:**
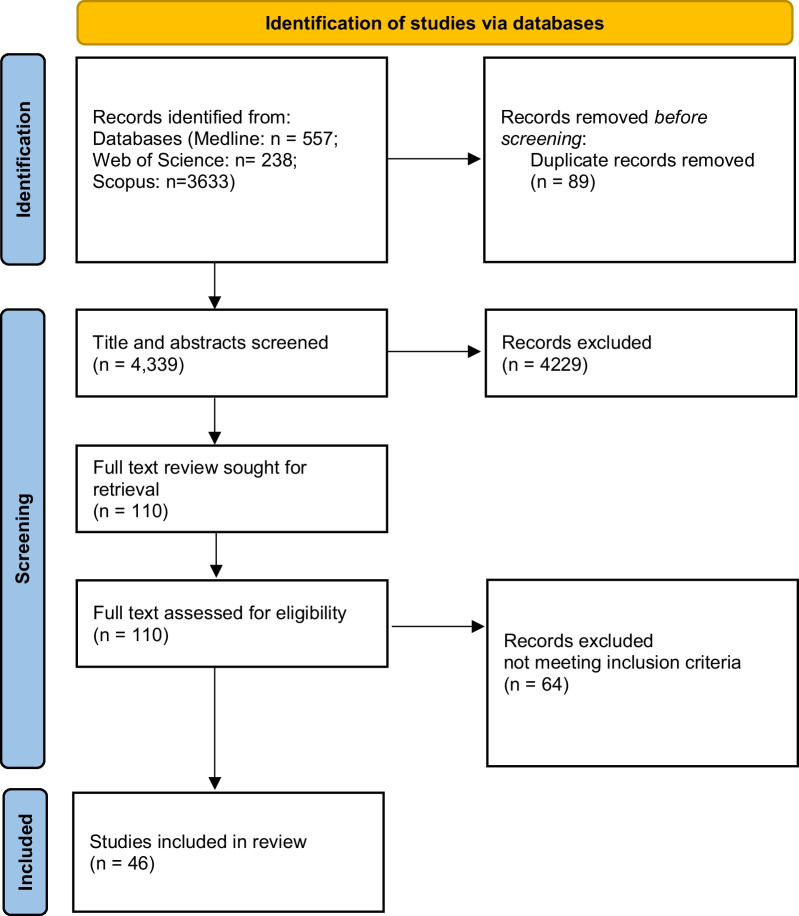
Flowchart of study inclusion

Covidence [19] was used to manage references, remove duplicates and review retrieved studies to include in the scoping review. Two reviewers assessed all titles and abstracts independently and determined articles to include for full-text review by consensus. Both authors bring diverse perspectives from their clinical and academic experience, one author having more than 20 years’ experience in CT clinical education as a radiographer (Lewis) and the other more than 20 years in radiation therapy education (Jimenez). Selected articles were screened in full text independently by two reviewers, and studies meeting the inclusion criteria were included in the scoping review. A third reviewer was not required to mediate decisions. This process is presented in Fig. 1.

Data charting was carried out using Covidence [19] and Microsoft Excel (Microsoft Corporations, Redmond, Washington). Relevant data were extracted from each of the included articles and synthesised into relevant topics. For publications with clinical or simulated data collection, aims of the study, geographic location of the study, data collection method, clinical setting, study sample were extracted. In addition, SEIPS domains within the results and discussion sections were identified for each of the included publications, guided by the descriptions in Table 1. Included studies were synthesised, and the results were organised by SEIPS model factors for articles that included each of the SEIPS domains. A formal assessment of quality of included studies was not undertaken, as it is not a typical feature of scoping reviews.

## Results

### Overview of included studies

**Fig. 2:**
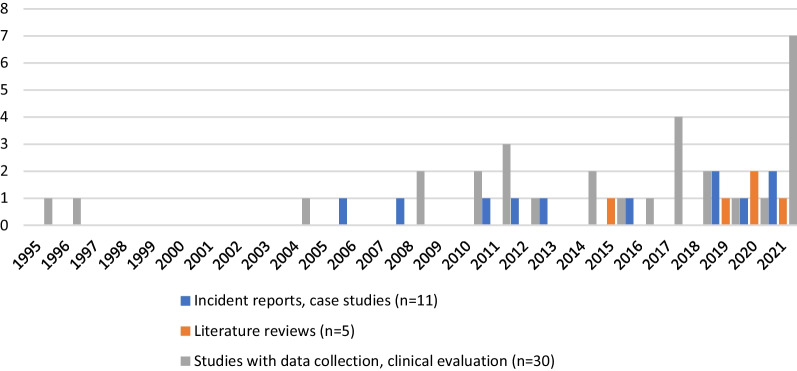
Included studies visualised by year of publication (*n* = 46)

A total of 46 articles were included in the scoping review. Included articles were published between the year 1995 and 2021, as detailed in Fig. 2. There were three main types of literature included: eleven incident report reviews or case studies [20–30], five literature reviews [2, 31–34], and thirty articles that employed data collection or clinical evaluations [35–64].

### Incident reports and case studies

**Fig. 3:**
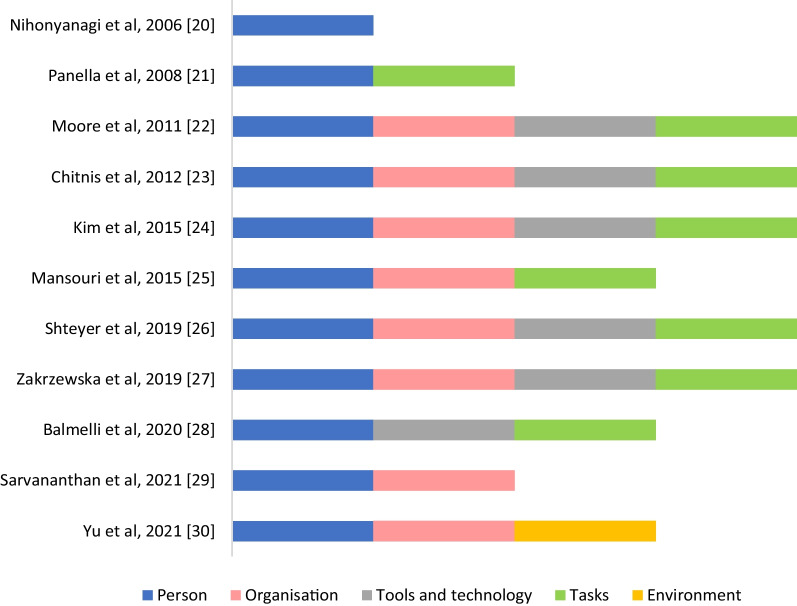
Summary of SEIPS domains within the included incident report reviews and case studies (*n* = 11). *Abstract only

Incident report reviews and case studies are summarised in Table 2, and the SEIPS domains included within the studies are shown in Fig. 3. These eleven publications provide an insight into IPC breaches that occur in the medical imaging environment. Within the SEIPS framework, the breaches most commonly related to the “person” Section domain, followed by “organisation” and “tasks” Sections. All but one incident report review/case study [20] had at least two SEIPS domains identified, with most publications integrating four domains (“Person”, “Organisation”, “Tools and technology”, and ‘Tasks’ Sections). Only one study included reference to ‘Environment’ [30].

**Table 2:** Study characteristic for included incident report reviews and case studies (*n* = 11)

First author, publication year	Overall study aim(s)	Clinical setting	Identified sample	Mechanism of infection/contributions to infections
Nihonyanagi et al., 2006* [20]	To report on multidrug-resistant Pseudomonas Aeruginosa isolated from clinical specimens in two patients	Portable X-ray device in internal medicine ward (Japan)	2 patients with multidrug-resistant Pseudomonas Aeruginosa	• Suspected that an individual radiographer neglected handwashing at the time of each patient’s X-ray procedure
Panella et al., 2008 [21]	To describe several cases of nosocomial HCV transmission	2 public hospitals, 1 private diagnostic centre. (Spain)	6 cases of HCV	• Possible source of transmission was a CT scan with contrast, health personnel manipulated the extension tube by disconnecting the tube from the patient first, and then from the equipment without changing gloves • No risk of blood contamination was detected from a contrast injector with automatic load from a 500-mL bottle that was shared by > 4 persons
Moore et al., 2011 [22]	To identify the source of incident HCV infection in a patient without identified risk factors, who had undergone myocardial perfusion imaging 6 weeks prior to diagnosis	Outpatient cardiology clinic. (USA)	2 potential source patients and 5 newly infected patients	• Evidence of HCV transmission among patients who had undergone myocardial perfusion imaging at the cardiology clinical on 2 separate dates • Transmission of HCV due to unsafe injection practices during myocardial perfusion imaging • Possibility that multi-patient use of vials occurred
Chitnis et al., 2012 [23]	To investigate an outbreak of bacterial meningitis at an outpatient radiology clinic, determine the source and implement measures to prevent additional infections	Radiology clinic (USA)	35 cases of bacterial meningitis	• Health care professional did not wear face mask; lapses in injection practice • Targeted education is needed among radiology health care professionals
Kim et al., 2013 [24]	To report on investigation and recommendations to control joint infections following arthrograms	MRI, outpatient radiology centre (USA)	7 cases (5 confirmed, 2 probable) identified, underwent procedure during a 1-week period	• No written procedures or documentation for infection control, aseptic-technique practices, medication preparation area cleaning/disinfection, staff training, or competency evaluations • Post-incident investigation observed that radiographers did not wash hands before preparation of injectable solutions; wore visibly soiled white coats, breaks in aseptic technique during preparation • Each vial of contrast media (labelled as ‘single dose’ by manufacturer), was re-entered with new syringes or needles multiple times for use on multiple patients
Mansouri et al., 2015 [25]	To describe multiyear experience in incident reporting related to MRI in large academic medical centre	MRI, large academic medical centre. (USA)	Infection control accounted for 0.4% of reported incidents	• Examples of incidents: patient was on tuberculosis precautions and staff member interacting with patient was not informed; needle stick injury while disposing needle; respiratory therapist detached ventilator tubing from patient on precautions for Methicillin-resistant Staphylococcus aureus, and handed it to staff member, saliva and fluid splashed in staff member’s face
Shteyer et al., 2019 [26]	To describe an outbreak of AHC in 12 patients	CT with contrast media. (Israel)	12 patients who received intravenous saline flush from a single multi-dose vial after intravenous contrast administration for a CT scan	• Probability of intravenous saline flush event resulting in transmission of Hepatitis C • Modelling suggested that microliter volumes of contaminated blood caused an outbreak of AHC during CT • Evaluation of the CT protocol and practices of the CT technicians identified the saline flush as the common source of exposure among the AHC patients
Zakrzewska et al., 2019* [27]	To analyse epidemiological situation of HCV in Poland in 2017	CT with contrast, hospital. (Poland)	HCV infection outbreak was registered: 8 patients, 291 exposed persons	• Common exposure was CT scan with contrast • Mechanism of infection transmission was not clearly identified, however, instructions for use, actions of the device and the activities of people who worked with device pointed to multiple deficiencies, on the part of the manufacturer and the hospital • From hospital, there was no risk assessment for the device used, no device decontamination procedures developed, and no regular staff training
Balmelli et al., 2020 [28]	To describe a case of HCV transmission from a chronic asymptomatic carrier to four patients through intravenous lines for contrast medium at an acute hospital	CT with contrast media, acute hospital. (Switzerland)	Patients (*n* = 14) HCV antibodies, presence of HCV RNA Four patients who underwent contrast-enhanced computed tomography (CT) scanning were infected with HCV from a contaminated multi-dose vial of Sodium Chloride	• Procedures that do not guarantee sterility: Routine insertion of a needle at the top of the vial to facilitate aspiration of the 0.9% sodium chloride solution was observed; the use of a 100-mL multi-dose vial of 0.9% sodium chloride to flush the intravenous lines of several patients before injection of contrast medium was found to be connected with outbreak. Approximately 10 mL of 0.9% sodium chloride was used for each patient, and a single vial lasted for eight to nine consecutive patients • Human error: It is hypothesised that a healthcare worker may have erroneously used the same syringe twice on a patient because of difficulties flushing the line or to set another one • Interviews with all involved healthcare workers revealed that none reported to undertake such behaviour, however, some of them did not consider the use of the same syringe twice on the same patient to be as wrong as using it on two different patients
Sarvananthan et al., 2021 [29]	To investigate the rates of incident reporting in a MID	Large academic health science centre. (Canada)	Hospital’s electronic incident report database. Incident report forms, * n* = 665 (July, 2018–July, 2019) Incident report rate (Discussion focused on top 4 incident types, which did not include infection control)	• Infection control was one of 14 incident type categories in a MID, accounting for 1.35% of incidents. Example: tuberculosis suspected in a patient, but patient arrived at MID without mask • Concerns about underreporting; need standardisation of incident reporting and reduced barriers to reporting, to improve the safety incident report system’s effectiveness • Weekly quality conversations and selecting a modality-specific safety representative
Yu et al., 2021[30]	Retrospective analysis of 2 cases of healthcare-associated COVID-19 transmission in 2 radiology departments, compared to the IPC practices in department, where no COVID-19 transmission occurred	Radiology department (China)	2 cases of health-care-associated COVID-19 transmission in 2 radiology departments	• Loopholes in IPC practices due to poor understanding of the COVID-19, need to establish isolation zones and additional sterilisation zones

^*^Abstract only; AHC: Acute Hepatitis C; CT: Computed Tomography; HCV: Hepatitis C virus; MRI: magnetic resonance imaging; MID: Medical Imaging Department

### Literature reviews

The topics researched in the included literature reviews focused on the COVID-19 pandemic [31, 33, 34] as well as pre- COVID-19 publications on general overview of healthcare-associated infections in the radiology department [2], and information on how to minimise infection transmission in the radiology department [32]. Some authors within the literature review publications associated the quality of IPC with staff knowledge and importance of standard precautions for all patients. They implied that transmission of infection organisms can occur at any stage of radiologic examinations.

### Key methods for evaluating IPC in medical imaging departments.

**Fig. 4:**
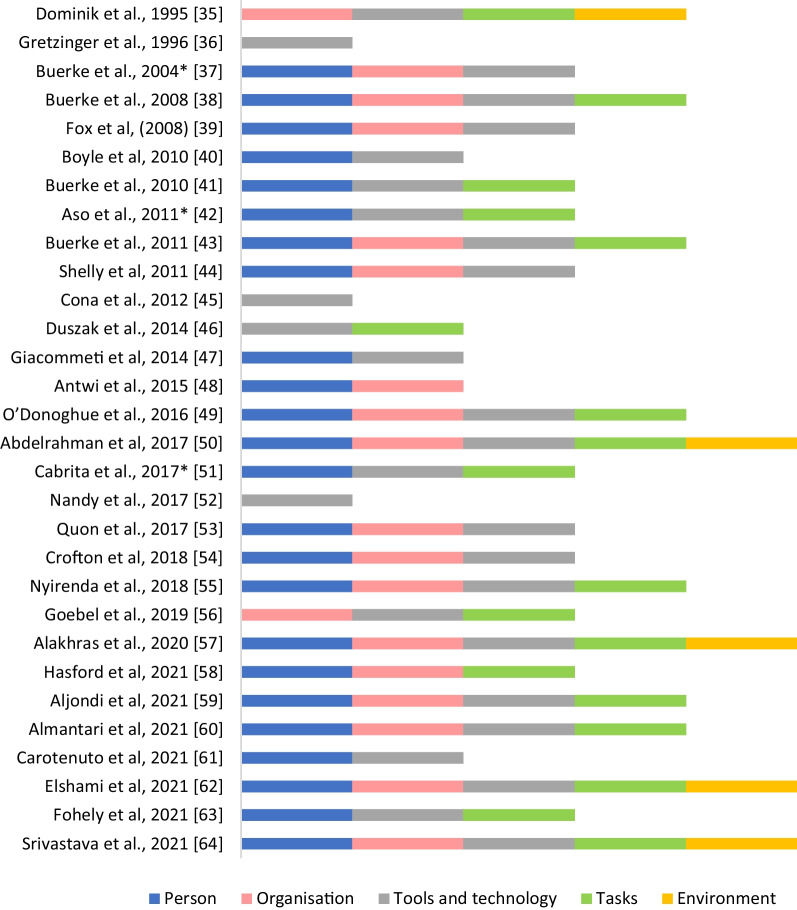
Summary of SEIPS domains within the included studies that employed data collection or clinical evaluations (*n* = 30). *Abstract only

For studies that included data collection (*n* = 30), there was a consistent trend in number of publications over the years, with a higher number of publications in the last five years. The most reported geographic location of the MID where data collection occurred (rather than authors’ affiliation) was Germany (*n* = 6). The type of data collection methods used most commonly were surveys/questionnaires [42, 43, 48–51, 53–55, 57–64], followed by microbial analysis [35, 37–44, 46, 47, 52, 54, 56], simulation studies [35, 36, 38, 52], direct observation or review of practice [44, 49, 51], timing [41, 43], and animal studies [45], as described in Table 3. The number of citing texts including each of the five SEIPS domains for studies that included data collection is shown in Fig. 4. The most common domains within these publications were “Tools and Technology” and “Person” Sections, with the least domain identified being ‘Environment’.

**Table 3:** Methods used in publications to explore IPC issues in medical imaging (*n* = 30)

First author, publication year	Overall study aim(s)	Setting	Sample	Data collection method						
				Microbial	Simulation	Survey	Review	Animal studies	Direct observation	Time
Dominik et al., 1995 [35]	To assess the potential risk of contamination and microbial multiplication in a non-ionic contrast medium when supplied in large volume containers and withdrawn in fraction over a certain period of time	Hospital (*n* = 9), Radiological departments (*n* = 1) (Germany)	Infusion bottles: * n* = 1000 (100 at each location)	•	•					
Gretzinger et al., 1996 [36]	To validate the integrity of one-way check valves for the delivery of contrast solution to multiple patients	(Canada)	Sprung valves and unsprung valves		•					
Buerke et al., 2004* [37]	To evaluate hygienic conditions using automatic injectors in MRI and CT during clinical routine	CT and MRI (Germany)	Medical devices and palms of technical and medical staff	•						
Buerke et al., 2008 [38]	To evaluate the risk of microbiologic contamination of the syringes of injectors used to administer contrast agent and saline solution in an experimental setting and clinical routine	Contrast-enhanced CT (Germany)	Experimental study, normal hygienic conditions: total * n* = 136 samples; Intensive hygienic prevention: total * n* = 136 samples Clinical study, imprints of palms for technical and medical staff: * n* = 44 each trial, surfaces (3 × PC keyboards, desktop of operational panel, telephone receiver, CT gantry, inner wall of CT tunnel, automatic injectors (button panel and syringes), and support pillows	•	•					
Fox et al., (2008) [39]	To investigate whether X-ray cassettes could be a source of pathogens capable of causing nosocomial infections in the hospital environment	Diagnostic imaging department (England)	X-ray cassettes: *n* = 40	•						
Boyle et al., 2010 [40]	To establish whether infection control measures were being undertaken sufficiently on lead rubber aprons within a diagnostic imaging department	Diagnostic imaging department (England)	Lead rubber aprons: *n* = 15	•						
Buerke et al., 2010 [41]	To evaluate the microbiologic contamination and time efficiency associated with routine clinical use of single-use pre-filled disposable syringes for contrast administration	(Germany)	Imprints of devices and the palms of hands of staff members were microbiologically analysed before the clinical investigation Single use of prefilled contrast syringes and saline syringes: *n* = 60, and single use of prefilled contrast syringes but multiple use of saline syringes for four injections or patients: *n* = 60	•						•
Aso et al., 2011* [42]	Not stated	Mobile X-ray system in emergency room, (Japan)	Radiological technologists: * n* = 22	•		•				
Buerke et al., 2011 [43]	To evaluate three different injection systems regarding microbial contamination, time efficiency, and user handling during clinical routine	Radiology, (Germany)	Patients; * n* = 825 Empty syringes, system A: *n* = 150; pre-filled syringes, system B: * n* = 150; Roller pump system C: * n* = 35 injections/day for 15 days	•		•				•
Shelly et al., 2011 [44]	To explore the potential risk to patients and healthcare workers of acquiring MRSA within a radiology department	Radiology Department (Ireland)	Environmental swabs: *n* = 125	•			•			
Cona et al., 2012 [45]	To verify whether a newly developed replaceable patient-delivery system may allow multiple uses of the system but without such risks	Belgium	Patient-delivery systems: * n* = 12	•				•		
Duszak et al., 2014 [46]	To quantity and characterise bacterial contamination of radiologist workstation	Inpatient and outpatient radiologist computer workstations in 2 teaching hospitals in 2 adjacent states. (USA)	Voice recognition dictation microphones: *n* = 7, and computer mice: *n* = 7. Toilet seats and door knobs as comparative samples	•						
Giacometti et al., 2014 [47]	To examine the level of microbiological contamination in the main radiology departments in Turin, Italy	12 radiology departments (2 private and 10 public), samples from X-ray tubes, control panels, radiographic cassettes and imaging plates Questionnaire to investigate use of PPE by radiology department chiefs (Italy)	In each Radiology department (*n* = 12) a total of 12 samples (3 for each of the four areas)	•		•				
Antwi et al., 2015 [48]	To assess the appropriate use of infection control by radiographers during radiological examinations in Ghana	3 hospitals (Ghana)	Qualified radiographers: *n* = 72			•				
O’Donoghue et al., 2016 [49]	To evaluate effectiveness on compliance of an intervention to improve awareness of hand hygiene	Radiography unit of district hospital (Hong Kong)	Radiographers: * n* = 76; nurses: * n* = 17; healthcare assistants: * n* = 9			•			•	
Abdelrahman et al., 2017 [50]	To evaluate radiographers’ knowledge of nosocomial infection control practices in Jordan	Educational, private, public hospitals (Jordan)	Radiographers: *n* = 100			•			•	
Cabrita et al., 2017* [51]	To evaluate whether radiographers wash their hands; to assess if radiographers use gloves properly; to check whether materials/equipment are disinfected when necessary; to assess the radiographers perception about hygiene standards	Public and private radiology departments (Portugal)	Radiographers			•			•	
Nandy et al., 2017 [52]	To test one way valves as a means of infection control used in medical device applications	(USA)	One-way valves: *n* = 5	•	•					
Quon et al., 2017 [53]	To evaluate workstation disinfection rates of hand hygiene of radiologists and trainees at shared workstations and assess the impact of education and reminder place-cards on daily habits	Tertiary care, academic institution (Canada)	Radiologists, fellows and residents			•				
Crofton et al., 2018 [54]	To investigate whether an awareness campaign will result in improvement in radiographers’ phone and hand hygiene practices	University hospitals (England)	Radiographers, pre-campaign:*n* = 36, post-campaign: *n* = 28	•		•				
Nyirenda et al., 2018 [55]	To determine the knowledge and practices of radiographers regarding infection control in radiology departments in government referral hospital in Malawi	Radiology departments in four Government referral hospitals (Malawi)	Radiographers: * n* = 62			•				
Goebel et al., 2019 [56]	To quantify the frequency of bacterial contamination of the injected contrast agent/saline solution by an automated contrast injection system, and to evaluate whether usage of a novel tube system can reduce it	MRI (Germany)	Patients: * n* = 101	•						
Alakhras et al., 2020 [57]	To assess the level of dental radiographers’ compliance with infection control measures and evaluate the factors affecting compliance	9 public hospitals and centres, 2 university affiliated hospitals, 100 private dental clinics/centres (Jordan)	Dental radiographers: * n* = 175			•				
Hasford et al., 2020 [58]	To assess the level of knowledge on SARS-COV-2 infection prevention, transmission and symptoms of COVID-19 among allied radiation medicine professionals	(Ghana)	Radiation medicine professionals: * n* = 145			•				
Aljondi et al., 2021 [59]	To assess the knowledge and practice of infection control for COVID-19 among healthcare workers in radiology departments in Saudi Arabia	Radiology departments (Saudi Arabia)	Radiographer, radiologic technologist, radiologist; *n* = 256			•				
Almantari et al., 2021 [60]	To assess the enforcement of infection control guidelines for patients with COVID-19 during medical imaging procedure and raise awareness f infection control in different hospital in Saudi Arabia	Saudi Arabia	Four hospitals in Saudi Arabia: *n* = 128			•				
Carotenuto et al., 2021 [61]	To identify factors important to patients for their return to elective imaging during COVID-19 pandemic	Elective MRI (USA)	Patients: *n* = 99			•				
Elshami et al., 2021 [62]	To investigate the response of the radiology workforce to the impact of COVID-19 on professional practice	(India, Middle East, North Africa)	Radiology staff: *n* = 903			•				
Fohely et al., 2021 [63]	To evaluate the overall knowledge of radiographers about IPC	Primary governmental hospitals in Southern West Bank and 3 private hospitals in the same area	Radiographers: * n* = 40			•				
Srivastava et al., 2021 [64]	To evaluate the perception and practice of IPC measures by Radiologists during pre-COVID-19 and present	Clinics, diagnostic centres, hospitals involved in performing ultrasound, reporting cross-sectional imaging, interventional radiology (India)	Radiologists: * n* = 152			•				

*Abstract only; COVID-19: Coronavirus 2019; MRI: Magnetic Resonance Imaging; CT: computed tomography; IPC: Infection prevention and control; PPE: personal protective equipment

### SEIPS Framework

To answer the research questions regarding domains of the SEIPS model that affect or influence IPC in medical imaging, the results of this scoping review are presented in five thematic sections: “Person”, “Organisation”, “Tools and technology”, “Tasks” and “Environment” Sections. Key topics within all included literature were identified, and examples from selected articles are provided, with the intention of addressing the five domains within the SEIPS framework.

### Persons

Health professionals in the included studies were radiological technologists or radiographers (referred to as radiographers herein), radiologists, medical staff/fellows and residents, nurses, and healthcare assistants. Most studies described hand hygiene related to staff working in MID and focused on specific aspects of hand hygiene, including interventions to improve hand hygiene practice [37, 49, 53, 54]. Two case studies reported on hand hygiene breaches by a radiographer. One resulted in bacterial infection of two patients [20] and the other in joint infections in seven patients after undergoing a magnetic resonance arthrogram [24]. Discussion relating to the importance of hand hygiene was significant in three literature reviews, published during the COVID-19 pandemic [31, 33, 34]. The common driver for hand hygiene in the included studies was the need for collaborative efforts of all radiology staff to assure provisions of high-quality and safe medical imaging services while safeguarding the health of the public, patients and healthcare professional, and this context was not exclusive to post-COVID-19 publications.

Five studies examined outcomes of an education intervention on hand hygiene [37, 38, 49, 53, 54]. O’Donoghue et al. reported that overall hand hygiene improved significantly following an education intervention. However, during their observations, it was reported that almost half of hand hygiene opportunities were missed by staff, indicating that further reinforcement of education may be needed. In the study by O’Donoghue et al., the effect of the intervention was evaluated two weeks after completion, demonstrating short-term compliance; however, long-term compliance was not evaluated [49]. Buerke et al. suggested that hygienic procedures should be evaluated using microbiology surveillance and unannounced evaluations of hygiene in CT departments [38]. Evaluation of an education intervention by Quon et al. focused on radiologists’ workstations in a Canadian tertiary care institution [53]. It was found that the percentage of radiologists who disinfected their workstations increased following education intervention; however, there were a small number of participants who indicated that disinfection was not necessary (9.5%). Attitudes towards whom should clean workstation varied, and trended towards ‘individual staff’ who used the workstations (52.7%), followed by ‘house-keeping staff’ (37.8%).

Studies that examined staff’s knowledge of IPC provided a range of insights from different professions. From a sample of 152 radiologists, Srivatstava et al. found that just over 50% of study participants had never attended a training session on IPC prior to the COVID-19 pandemic, and the majority (86%) indicated that their knowledge on IPC had improved during the COVID-19 pandemic [64]. Another study [57] described poor compliance with infection control practices among dental radiographers, and compliance was inconsistent across demographic factors and types of infection control practices. The reason for variation among demographic groups was reported to be unclear; however, a general trend was that less experienced radiographers were more likely to follow IPC procedures, and those that performed fewer dental radiographs, or radiographs in private clinics, were more likely to implement IPC procedures. An increased awareness of IPC in radiographers with longer working experience was also found in a study by Fohely et al., based on a sample of 40 radiographers [63].

The case studies included in this review provided insights into the consequences of staff’s IPC knowledge, practice breaches and use of equipment. Sarvananthan et al. reported that infection control was one of 14 incident type categories in a MID, accounting for only 1.35% of incidents [29]. The transmission of Hepatitis C was described in studies relating to contrast-enhanced CT procedures [22, 26–28]. For example, in the study by Balmelli et al., breaches in safe injection practices were attributed to vial contamination, yet unsafe practices were not self-reported by staff who were interviewed [28].

One study provided a patient lens to the concept of IPC in MID. Carotenuto et al. surveyed patients whose elective MRI procedures had been delayed due to the COVID-19 pandemic. It was identified that patients considered MRI to be safe to visit and prioritised staff practices, such as using masks, as an important IPC factor [61].

### Organisation

Studies that evaluated or discussed the relationship between medical imaging organisational factors, such as organisation culture, staffing number, interdisciplinary communication and collaboration and incident reporting were not evident in the included literature. However, Source control, early detection of suspected COVID-19 cases followed by immediate isolation of such patients, establishment of efficient central coordination between hospital IPC centre, and the radiology department were highlighted by Srivastava et al., [64]. It was also noted that 54% of radiologists had not had any formal IPC training from their organisation prior to the COVID-19 pandemic [64].

Other organisational barriers reported in the literature included limited information available for health professionals. For example, Quon et al. indicated that at one institution, no current protocol mandating workstation disinfection was present, and further all participants in their study indicated that they had never received instruction on how to properly disinfect their workstation, leading to individual’s personal discretion when making decisions about disinfection practices in the radiologist workstation setting [53].

One study [29] showed that their results support projects to investigate ways to improve patient safety culture within medical imaging. One way to foster an improved patient safety culture was by promoting group discussions and shared accountability in advocating for safe care, for example, by conducting weekly quality conversations and selecting a modality-specific safety representative [29]. Further, concerns about underreporting IPC, standardisation of incident reporting, and reduced barriers to reporting will be essential in improving the effectiveness of the current safety incident report system.

### Tools and technology

IPC relating to equipment used in medical imaging included bacterial infection on computed radiography consoles on Hospital Information system/Radiology Information System terminals, which were not wiped down and cleansed as part of routine cleaning, and disinfection [42]. Regular disinfection of all surfaces that patients may be in contact with, and the use of dedicated portable X-ray or diagnostic equipment was also recommended [64].

It was also considered that medical devices/technologies, such as contrast injectors, required a level of skill to be used and a suitable environment in which to operate and maintain the equipment. A study by Shteyer et al. reported on an outbreak of acute Hepatitis C (AHC) among 12 patients who received intravenous saline flush from a single multi-dose vial after intravenous contrast administration for a CT scan in a medical centre in Israel [26]. From the investigations conducted, it was identified that the saline flush was the common source of exposure among the AHC patients. The study highlighted the importance of using extra care to ensure that no contamination occurs, since even microliter amounts of infected blood diluted in saline can lead to Hepatitis C virus (HCV) outbreaks, and further emphasises the need for prevention strategies and vaccines to eliminate HVC transmission.

The medical imaging setting also comprises of reporting rooms, where radiologists and other staff view and report on images. One study [53] reported on microbial contamination in radiology reporting workstations, which are often shared by staff, and where people may interact with up to 4 different workstations per shift. Hand sanitiser units were mostly considered readily available within the environment, situated within walking distance, yet could be improved by placing within each reading room. Having place cards on desks to remind radiologists of disinfection were used. In addition, each examination room had a handwashing sink and an alcohol-based hand rub dispenser.

Personal protective equipment (PPE) was also highlighted as key tool relating to IPC in MID. The importance of masks and/or gloves was highlighted in many studies [49–51, 57, 59, 61, 64], including the lack of resources or breaches of PPE as major challenges to IPC in MID [21, 23, 29, 51, 55, 62], including identified detriments to patients’ health [21, 23, 29].

### Tasks

For radiographers and other staff working in the medical imaging environment, procedures are usually multi-step and may involve positioning and stabilising the patient for imaging, operating equipment and if required, cannulating and/or connecting the patient to a contrast media injector. IPC steps are integrated into these tasks, and evidence of sub-optimal hand hygiene was reported in the literature. For example, hand-hygiene opportunities before and after patient contact were missed in 78% of occasions, as reported in an observation study [49]. Workload and time pressures may negatively impact IPC in medical imaging settings, and this was highlighted by a study evaluating five different aspects of IPC [57]. In addition, time and resources needed during pre- and post-CT scans were amplified during the COVID-9 pandemic [34].

Examples of contamination in the medical imaging setting related to infection of patients with Hepatitis C [22]. Examples included in the case study identified high-risk tasks and sub-optimal care taken undertaken in the process of administering saline flush from a multi-use larger saline bag after the injection of radioisotopes and possible re-use of needles between pharmaceutical injection and saline [22].

### Environment

Included articles reported on environmental modifications to the MID in response to the COVID-19 pandemic [24, 31, 34, 64]. This included triage stations at entry of healthcare facility and visual warnings, such as IPC posters and signs. Reminders relating to IPC located visibly within the environment were also highlighted, for example using visible place cards relating to cleaning of computer workstations [34]. When considering modifications to the physical environment, results from a survey conducted by Srivatstava et al. identified that radiologists considered the radiography table and CT scanner as the most likely areas of the radiology department for pathogen exposure [64]. Hence, IPC practice focused on the cleaning and disinfecting these hard surfaces and areas. Air quality was also mentioned by Srivatstava et al., suggesting patients should have adequately ventilated rooms [64]. In a retrospective analysis, poor understanding of the COVID-19 disease was attributed to healthcare-associated COVID-19 transmission in 2 radiology departments in China [30]. Modifications to the environment, for example establishment of isolation and sterilisation zones, were recommended to meet the demands placed by the disease transmission mechanism.

## Discussion

This scoping review identified work system factors using the SEIPS model that influence the practice of IPC in MID. Many publications reported on multiple levels of engagement, and as such, all five domains of the SEIPS model were identified in the included studies. The SEIPS model provides insights of the entire system, which may be used to uncover the causes of errors and near misses relating to IPC in MID, as described in Table 2 for the eleven included case studies and incident reports.

Medical imaging environments are not generally categorised as sterile zones, and MID in hospitals experience ongoing interactions between patients and staff, and often high staff and patient rotation. During medical imaging procedures, a healthcare professional will follow a few procedural steps, in which active participation of IPC guidelines and best practice is required. The SEIPS framework suggests that when analysing challenges of IPC, an individual’s performance should be examined for the purpose of re-designing work systems to reduce barriers to human performance [10].

The scoping review provided insights about the level of knowledge of IPC and specific attitudes to IPC and practices of IPC for staff working in MID. Hand hygiene was the focus of most published studies. Hand hygiene is a behaviour associated with individuals, which includes an inherent component that is a natural self-protecting in response to visibly or conceptually contaminated hands [65]. In contrast, reinforcement by organisations may be required for individuals to adopt practices of hand hygiene that are not instinctive. Reinforcement of the importance of elective components of hand hygiene, compared to inherent components, links to education and monitoring of the behaviour, which can be categorised as the organisation’s responsibility. Whilst education interventions were shown to strengthen health professionals’ hand hygiene and IPC practice in five studies [37–39, 53, 54], evidence about the monitoring or pre-requisites for medical imaging professionals’ willingness to use best practice in IPC is lacking from the identified literature. In addition, the decision for individuals to perform hand hygiene is influenced by both automatic and conscious processes. In observational studies that identified breaches in performance, data were not collected about reasons for non-compliance (for example, asking staff about missed opportunities immediately following the event), suggesting the need for further research in this area.

For hand hygiene, tools and the availability of resources are important factors [66]. For example, one study described that improvement of the location of alcohol-based hand rub at point of care, facilitated decontamination of hands. The concept of the environment also links to the concept of IPC behaviours as being an essential part of the professional role, and creating an environment that encourages positive behaviour. Importantly, for staff to adhere to preferred IPC behaviours, they require an environment that supports these actions. It is reported that workload and time pressure may negatively impact IPC in healthcare settings [66], and this was supported in one study included in this review, which noted that higher patient caseload could be associated with lower compliance with IPC [57].

Environmental issues with IPC published since the year 2020 mainly focused on modifications to the medical imaging environment in response to the COVID-19 pandemic. This integrated the concern for increased risk of radiographers contracting COVID-19 due to routine diagnosis, assessment and monitoring of COVID-19 patients using medical imaging procedures [34]. A focus on disinfection of imaging and treatment beds, equipment, and considerations of air quality were reported [64]. Adaptation, as a response to the complexity of health care in pandemic times, was evident in the literature. These adaptations were discussed in the form of adjustments that people and organisations needed to make to conduct their work safely, such as being spatially aware and procedurally orientated when moving about medical imaging rooms and settings, considering that infection risk (as is the case with COVID-19) is not often visual, but more broadly related to shared environments.

Sub-optimal task performance at each of the stages of medical imaging procedure was considered to place staff member or patients at risk. Case studies identified in the scoping review provided insights into breaches, and breaches related to all SEIPS domains. The transmission of HCV was described in studies relating to contrast-enhanced CT procedures and nuclear medicine studies [21, 22, 26–28]. For example, in the study by Balmelli et al., breaches in safe injection practices were attributed to vial contamination. Interestingly, interviews with healthcare workers revealed that no one reported that they had undertaken such behaviour [28]. This confirms the limitation of interviews and self-reported data collection for IPC practice, where desirable responses are observed and there is fear of litigation. This is supported by previous studies acknowledging the under-reporting of errors in MID [67]. Knowledge-based tasks require providers to problem-solve when faced with new situations or reinforce best IPC practices at intervals to ensure up-to-date knowledge. Knowledge-based errors occur when a health professionals’ knowledge is incomplete or incorrect [34], and the health professional does not know what they must know or where standards may have changed in response to new evidence.

The scoping review identified that IPC studies in the medical imaging setting include prospective studies using self-reported surveys and microbial analysis as the most used study designs for data collection, followed by mixed methods study designs involving simulation and observation. Case studies and incident reports focus mostly on microbial measurements and viral analysis. IPC knowledge, attitude and practice were mostly captured in survey-based studies. Whilst these methods can be useful, they provide heterogeneous data, which incorporates social desirability bias, and is possible that participants report that they perform certain behaviours more or less than they do. In addition, data from these studies do not contribute to improved understanding relating to reasons why an IPC behaviour is performed or not performed. Combining staff interviews with observation may provide a more accurate view of compliance. The evaluation of IPC breaches was reported in the case studies, yet these focused on the task, tools and technologies, rather than the operator, organisation or environment, so it is not clear what was the influence on personal behaviour. In addition, details of the IPC breach were lacking. In most cases, tasks that involved IPC breaches from the included studies related to the skills-based cognitive domain, which resulted from failure to carry out best practice by lack of attention or when actions are omitted (e.g. missed opportunities for hand hygiene). It was not evident from the literature if these skill-based errors occurred due to a specific situation, for example time pressures, specific type of patients, or multi-tasking. In addition, most IPC failure data emerged from self-reported surveys, which as previously discussed, may not be the optimal source of this type of data.

### Implications for practice and future research

Evaluation or discussion of the relationship between medical imaging organisational factors, such as organisation culture, staffing number, interdisciplinary communication and collaboration and incident reporting, is generally evident in types of studies not included in this scoping review, for example, commentary articles released in response to the COVID-19 pandemic [4–8]. In these publications, the motivation to provide a safe working environment for medical imaging staff and patients was strongly emphasised by leaders within MID. Culture is developed over time by leaders who set a vision for safety as a priority of care, and who manage change effectively. Leaders must build trust because it is a cornerstone of a culture safety [4]. In addition, it can be considered that without trust, health professionals will not discuss near-misses, responses should adopt a non-punitive approach where health professionals are not blamed, but rather, the system is examined to find ways to improve task, technology, environment, or communication. Commentary articles suggest that staff shortage, lack of resources and lack of communication can be associated with IPC challenges in the medical imaging setting.

In the future, it will be important to undertake research to better understand the current culture, teamwork environment, and usability of the technology and processes involved that may challenge IPC in MID. In addition, observational research methods are currently under-reported and may best serve to identify underlying systems. Interestingly, none of the papers that investigated education interventions to improve knowledge and behaviour in IPC applied a theoretical framework or looked at long-term outcomes in staff knowledge or behaviour, nor patient outcomes or monitoring of outcomes over time. Hence, it is unknown whether results of current education are long lasting. There is a need for future studies to prospectively implement and evaluate IPC education and training in MID to ascertain the long-term benefits and role of monitoring. Finally, further research could focus on contrast media in CT imaging, considering that these were the main source of reported adverse outcomes for patients in the included studies.

The important role of radiographers in performing mobile imaging in high care environments such as ICUs, with strict IPC guidelines, is recognised. However, in the case of this scoping review, mobile radiography was excluded, as the protocols and unique needs of nursing and surgical areas outside radiology departments are generally managed by external health staff, such as Nursing Unit Managers. We suggest that future research and education are also required for imaging that takes place as a mobile examination (such as imaging in ICU) to ensure radiographers understand the unique requirements of these high care environments, such as patients and staff working in isolation and barrier areas.

### Limitations

Limitations may exist in this scoping review due to the review process and design. The search was limited to English-language publications, and the body of literature related to this topic may also be subject to publication bias, as negative outcomes are less likely to be published. It is possible that some applicable studies were missed due to incomplete search terms or unintended reviewer bias. The inclusion of abstracts in the review limited the information available to be extracted due to the concise nature of this type of publication. Potential sources of heterogeneity in our scoping review are different study populations, diverse geographical regions and study designs; consequently, the results should be interpreted with caution. Diversity in geographical regions needs to be taken into consideration when interpreting results from this study, as practice and availability of resources is known to vary between different countries, where work conditions, infrastructure and healthcare systems are diverse.

## Conclusion

IPC in the medical imaging setting would benefit from a systems approach, linking the five components: “person”, “organisation”, “tools and technologies”, “tasks’ and ‘environment” Sections. The identified literature supports the interrelated nature of the five components and influence on one another; further evidence is required to establish how changes to one component affect the others. To make solid inferences and suggest recommendations for practice and policy, systematic reviews and focused IPC studies in the medical imaging domain are suggested. Future studies also need to address the role of education and monitoring of IPC compliance in the clinical setting, to increase the body of knowledge regarding the long-term outcomes of education interventions.

## Data Availability

All data generated or analysed during this study are included in this published article and its supplementary information files.

## Abbreviations

AHC: Acute Hepatitis C
COVID-19: Coronavirus-19
CT: Computed tomography
HCV: Hepatitis C virus
IPC: Infection Prevention and Control
MID: Medical Imaging Departments
MRI: Magnetic resonance imaging
PRISMA: Preferred Reporting Items for Systematic Reviews and Meta-analysis
SEIPS: Systems Engineering Initiative for Patient Safety
